# Predicting radiographic outcomes of vertebral body tethering in adolescent idiopathic scoliosis patients using machine learning

**DOI:** 10.1371/journal.pone.0296739

**Published:** 2024-01-12

**Authors:** Ausilah Alfraihat, Amer F. Samdani, Sriram Balasubramanian

**Affiliations:** 1 School of Biomedical Engineering, Science and Health Systems, Drexel University, Philadelphia, PA, United States of America; 2 Hashemite University, Zarqa, Jordan; 3 Shriners Hospitals for Children, Philadelphia, PA, United States of America; Columbia University Vagelos College of Physicians and Surgeons, UNITED STATES

## Abstract

Anterior Vertebral Body Tethering (AVBT) is a growing alternative treatment for adolescent idiopathic scoliosis (AIS), offering an option besides spinal fusion. While AVBT aims to correct spinal deformity through growth correction, its outcomes have been mixed. To improve surgical outcomes, this study aimed to develop a machine learning-based tool to predict short- and midterm spinal curve correction in AIS patients who underwent AVBT surgery, using the most predictive clinical, radiographic, and surgical parameters. After institutional review board approval and based on inclusion criteria, 91 AIS patients who underwent AVBT surgery were selected from the Shriners Hospitals for Children, Philadelphia. For all patients, longitudinal standing (PA or AP, and lateral) and side bending spinal Radiographs were retrospectively obtained at six visits: preop and first standing, one year, two years, five years postop, and at the most recent follow-up. Demographic, radiographic, and surgical features associated with curve correction were collected. The sequential backward feature selection method was used to eliminate correlated features and to provide a rank-ordered list of the most predictive features of the AVBT correction. A Gradient Boosting Regressor (GBR) model was trained and tested using the selected features to predict the final correction of the curve in AIS patients. Eleven most predictive features were identified. The GBR model predicted the final Cobb angle with an average error of 6.3 ± 5.6 degrees. The model also provided a prediction interval, where 84% of the actual values were within the 90% prediction interval. A list of the most predictive features for AVBT curve correction was provided. The GBR model, trained on these features, predicted the final curve magnitude with a clinically acceptable margin of error. This model can be used as a clinical tool to plan AVBT surgical parameters and improve outcomes.

## Introduction

Anterior Vertebral Body Tethering (AVBT), a novel growth-friendly modulation technique, is used to guide spinal growth to correct scoliosis deformity via the application of the Heuter-Volkmann principle [1–5]. AVBT is used for skeletally immature adolescent idiopathic scoliosis (AIS) patients with thoracic curves measuring 30 to 65 degrees that continue to progress despite bracing. Since its approval by the US Food and Drug Administration (FDA) as a Humanitarian Use Device in 2019, AVBT has shown promising short- and mid-term results in several clinical studies [6–10]. However, the predictability and reliability of spinal growth modulation in vivo remain uncertain and hinge on a complex interplay of factors, including magnitude of the curve, expected growth remaining, and the surgical parameters [7–21].

Several studies have attempted to improve the predictability of AVBT outcomes. Yucekul et al [22] performed a retrospective analysis of demographic and radiographic data in 38 AIS patients who underwent Vertebral Body Tethering (VBT) surgery. They employed various methods, including Risser, Sanders Simplified Skeletal Maturity Staging (SSMS), Thumb-Ossification Composite Index (TOCI), Combined Hand Maturity Scale (CHMS), and Cervical Vertebral Maturity (CVM), to determine the patient’s skeletal maturity. Subsequently, they used regression models to assess longitudinal growth and growth modulation. They found that the Growth Modulation Scale (GMS), created by integrating CVM with CHMS, had the highest predictive ability for growth modulation years after VBT surgery [2–4]. Although the GMS demonstrated a strong correlation with longitudinal growth and growth modulation, external validation of the GMS in larger cohorts is necessary, and staging of the CVM presents challenges.

Mandel et al [23–25] published a series of studies on the use of statistical and machine learning models for predicting the surgical outcomes of Anterior Vertebral Body Tethering (AVBT) procedures in adolescents with idiopathic scoliosis. They developed statistical frameworks and predictive networks that use 3D spine reconstructions from follow-up radiographs to predict the surgical outcomes of AVBT. The models are based on subject-specific correction trajectories, temporal dynamics in curve progression, and clinical parameters. Although the results showed that the models were accurate in predicting the surgical outcomes with errors of around 2.1 degrees in main curve magnitude, the process of reconstructing 3D models from 2D radiographs is complicated.

The use of a 3D finite element methods (FEM) to simulate spine correction has emerged as a promising approach for predicting surgical outcomes in patients with scoliosis [3–5, 26, 27]. Studies have shown that transforming preoperative models of scoliotic patients to the intra-operative lateral decubitus position significantly corrected the thoracic spine [28]. The concept of FEM has been further explored in the context of growth dynamics by analyzing the internal stress distributions within the intervertebral discs and growth plates of compressive growth modulation devices [29, 30]. These studies have demonstrated the capability of reducing asymmetrical loading on growth plates and the possibility of progressive growth correction. However, integrating several physiological and clinical parameters to modulate the stresses applied to the epiphyseal plate remains a challenge [29, 30]. Despite the potential of biomechanical models to reproduce surgical outcomes in AVBT, they are computationally complex and require further advancement [31].

The current limitations of AVBT outcome prediction models highlight the need for developing improved prediction models for AVBT procedure. Hence, this study aims to develop a machine learning-based prognostic tool that utilizes a combination of clinical, radiographic, and surgical parameters to predict short- and mid-term corrections achieved in AIS patients who underwent AVBT surgery.

## Methods

### Inclusion criteria for patients’ selection

Institutional review board approval (Protocol #PHL2102R) was obtained by western institutional review board. Before accessing the database, all data were fully anonymized and as the data is retrospective the western institutional review board waived the requirement for informed consent. A retrospective analysis was conducted on pediatric patients from the Shriners Hospitals for Children database from 2011 to 2020. The selection of patients was based on the following inclusion criteria: (1) primary diagnosis of AIS with any Lenke classification, (2) patients who were under 21 years of age, (3) patients who underwent VBT treatment for scoliosis, and (4) patients with First Erect (FE) follow-up and at least one follow-up visit after their initial VBT treatment, including preoperative radiographic images (posteroanterior (PA), anteroposterior (AP), lateral, and side bending) taken using X-ray, CT, or EOS imaging.

### Data collection

The data collected for this study included demographic, radiographic, and surgical features associated with curve correction. A total of twenty continuous variables and seven categorical variables were analyzed. The complete list of features is provided in Table 1.

**Table 1:** Features collected from patients’ radiographs and clinical records.

Demographic (n = 3)	Radiographic (n = 20)	
Age at surgery	Major Cobb	Coronal Balance
Age at visit	Major Cobb tether	Shoulder Balance
Gender*	Lumbar Cobb angle	L4 tilt
**Surgical (n = 4)**	Thoracic Kyphosis	Trunk shift
First Erect Cobb angle	Lumbar Lordosis	Bending Proximal Thoracic
Upper Instrumented Vertebrae (UIV) *	Proximal Thoracic Kyphosis	Bending Thoracic
Lower Instrumented Vertebrae (LIV) *	Mid lower thoracic kyphosis	Bending Lumbar
Number of Instrumented Vertebrae (NIV) *	Thoracolumbar kyphosis	Curve Flexibility
	Thoracic curve apex *	Sanders Maturity Stage *
	Lumbar curve apex *	Major curve apex *

Note: Variables marked with an asterisk (*) indicate categorical variables.

### Demographic features

Based on the inclusion criteria, 91 pediatric patients with AIS (11 males and 80 females) were selected for the current study. Demographic information such as gender, age, and the time elapsed between follow-up visits was collected.

### Radiographic features

**Fig 1:**
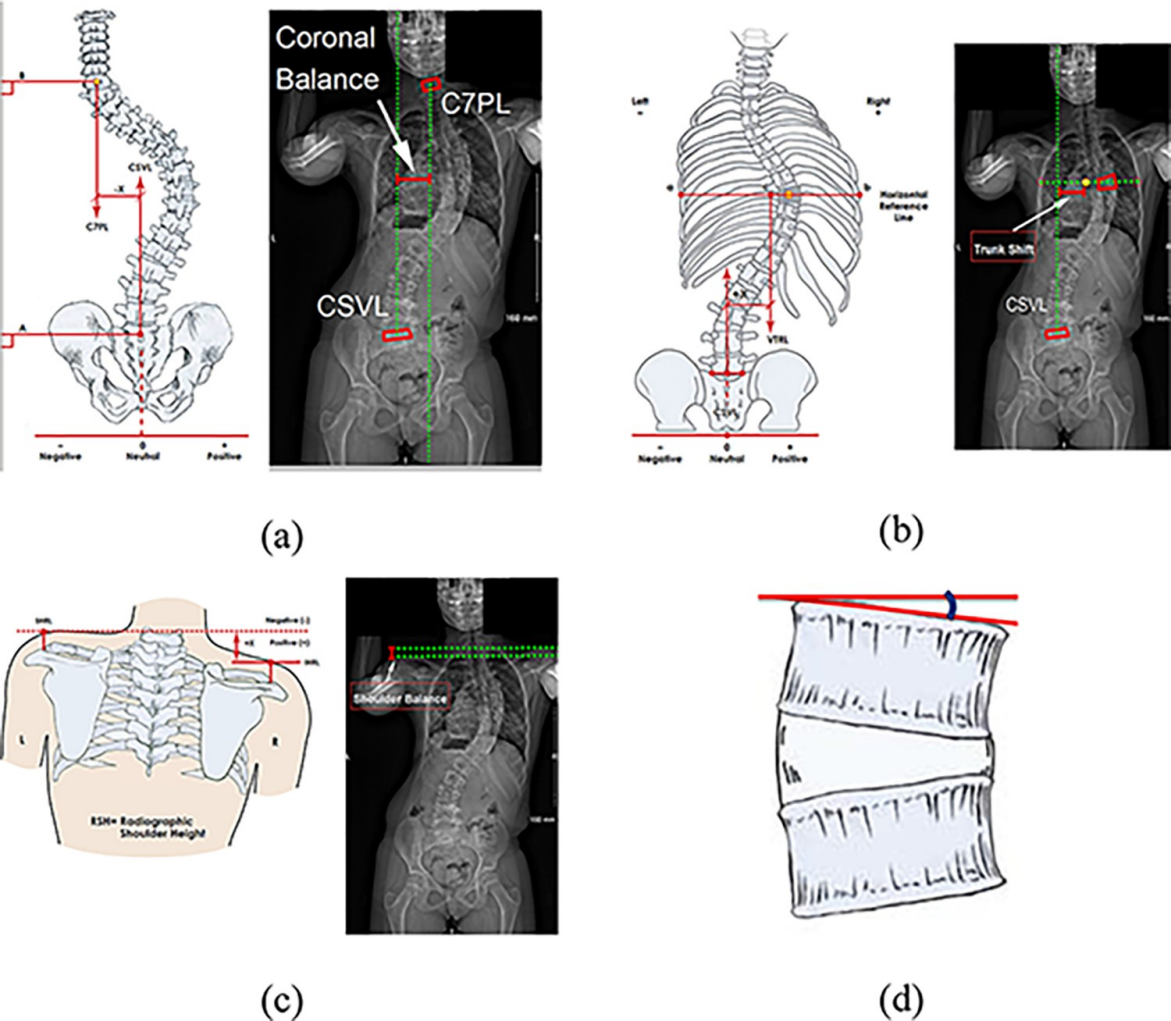
Features extracted from frontal radiographs (adapted from [33]). (a) coronal balance was measured as the shortest horizontal distance between the vertical line which passes through the centroid of the seventh cervical vertebrae named as C7 plumb line (C7PL) and the vertical line through the centroid of the first sacrum vertebrae named as Central Sacral Vertical Line (CSVL) (b) to extract the trunk shift measurement, the apical thoracic vertebra is identified, and a horizontal line (ab) is drawn through its centroid. The trunk shift is then measured as the shortest distance between the vertical line through the midpoint of the line (ab) and the CSVL (c) shoulder balance was measured as the shortest vertical distance between two corresponding points on the left and right clavicle bones (d) L4 tilt is the angle measured between the superior endplate of the fourth lumbar vertebrae (L4) and the horizontal.

**Fig 2:**
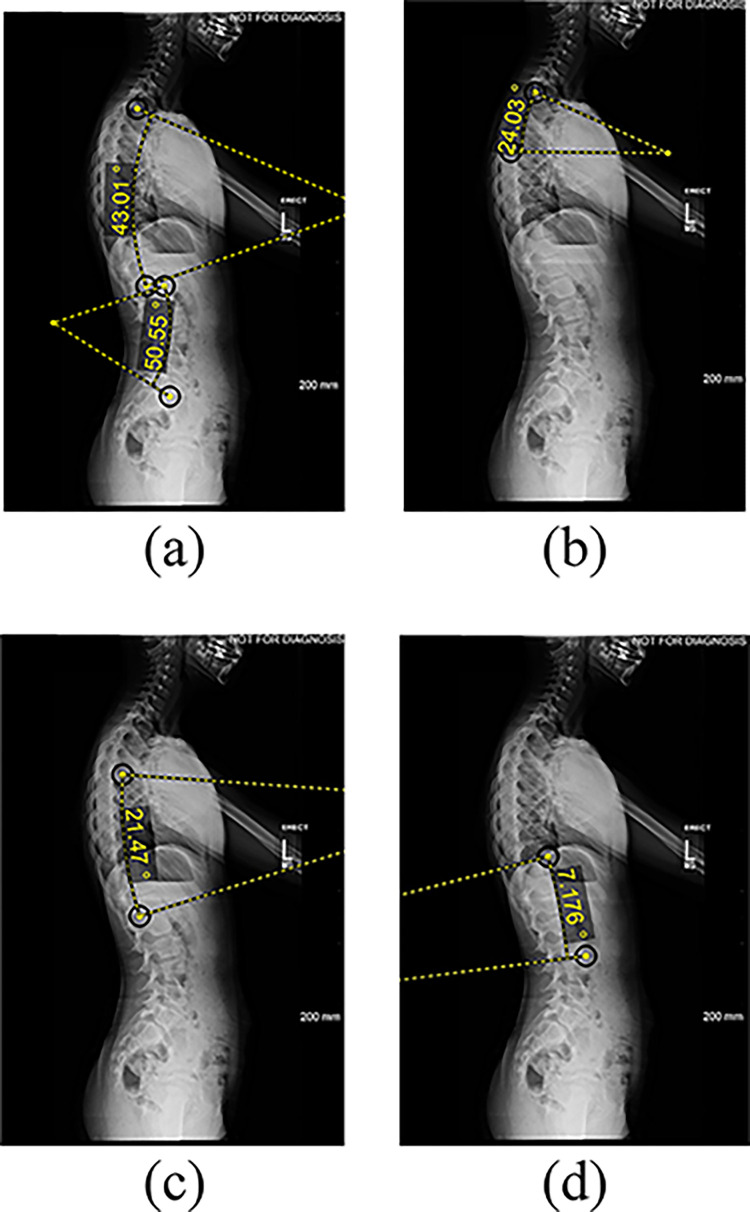
Features extracted from lateral radiographs. (a) thoracic kyphosis and lumbar lordosis (b) proximal thoracic kyphosis (c) mid lower thoracic kyphosis (d) thoracolumbar lordosis angle measured between the superior endplate and the inferior endplate of the vertebral levels: T2-T12, L1-L5, T2-T5, T5-T12, and T12-L2, respectively.

The radiographic features of the 91 pediatric patients with AIS were analyzed in this study. Standing (PA or AP, and lateral) and side bending spinal radiographs were obtained from all patients at six visits: preoperative, first standing, one year, two years, five years postoperative, and at most recent follow-up. Previously published methods for landmark point (LMP) selection and measurement extraction were used in this study [32]. From the frontal radiographs, four radiographic features were extracted, including coronal balance, trunk shift, shoulder balance, and the tilt of the fourth lumbar vertebra (L4) (as shown in Fig 1) [33]. Additionally, five features were extracted from the lateral radiographs, including kyphosis, lordosis, proximal thoracic kyphosis, mid-lower thoracic kyphosis, and thoracolumbar lordosis (as shown in Fig 2). These angles were measured between the superior and inferior endplates of the vertebral levels T2-T12, L1-L5, T2-T5, T5-T12, and T12-L2, respectively. As this study was retrospective, the Cobb angle at the first erect for the purpose of training and testing the machine learning model was directly measured from the postop radiograph. Since this model would be used for AVBT surgical planning, where the actual post-operative first erect Cobb angle measurement will not be available, the Cobb angle at first erect can be estimated from the preop bending radiograph using a previously published equation [34]. Where: *First erect major Cobb angle (in degrees)* = 7.5 + 0.65 × Fulcrum−Bending Cobb angle (in degrees). Fulcrum-Bending Cobb angle is defined as the reduced Cobb angle when the patient performs a lateral bend towards the convexity.

### Surgical features

Surgical features were extracted from the postoperative radiographs of the patients. The surgical information collected included one continuous feature, the magnitude of the curve that was instrumented in the first standing radiograph after the AVBT procedure, and three categorical features namely, Upper Instrumented Vertebra (UIV), Lower Instrumented Vertebra (LIV), and Number of Instrumented Vertebrae (NIV).

### Sequential Backward Floating Selection (SBFS)

To enhance prediction accuracy, reduce model complexity, and eliminate correlated or redundant features, the study utilized the Sequential Backward Floating Selection (SBFS) method. This method was implemented using mlxtend (version 0.18.0 for python 3.6) [35] as part of a pipeline that included cross-validation from Scikit-learn (version 0.21.3 for python 3.6) [36]. The SBFS method began with all features listed in Table 2 and evaluated the regression between each feature and the target (postoperative major Cobb angle). Based on the mean absolute error (MAE) performance metric, the feature that minimized the MAE after removal was excluded and the model was refitted. A conditional inclusion step was added after each removal to determine if the MAE was minimized after a feature was re-added. The subset of features with the minimum MAE was chosen as the input for the predictive model.

**Table 2:** Indices of input features to SBFS.

Index	Feature
0	Major Cobb Angle
1	Preop secondary Cobb angle
2	First erect major Cobb angle
3	Preop thoracic curve apex
4	Preop lumbar curve apex
5	Major curve Apex
6	Age at Surgery
7	Age at visit
8	Sanders stage
9	Gender
10	Coronal balance
11	Shoulder balance
12	L4 tilt
13	Trunk shift
14	Thoracic Kyphosis
15	Preop proximal thoracic kyphosis
16	Mid lower thoracic kyphosis
17	Thoracolumbar kyphosis
18	Lumbar lordosis
19	Preop proximal thoracic bending curve
20	Preop thoracic bending curve
21	Preop lumbar bending curve
22	UIL tethered levels
23	LLI tethered levels
24	NLI tethered levels
25	Preop tethered Cobb angle
26	Flexibility

### Machine learning model selection and optimization

The final major Cobb angle post-AVBT (at first erect time-point) in pediatric patients with AIS was predicted by utilizing a dataset of the most predictive features. The dataset (for all patients) was randomly split into training (80%) and testing (20%) datasets, and four widely-used machine learning models, namely Random Forest (RF) [37], Gradient Boosting Regressor (GBR) [38], Support Vector Machine (SVM) [39, 40], and Artificial Neural Network (ANN) [41, 42] were trained using a 5-fold cross-validation with the scikit-learn library (version 0.21.3 for python 3.6). To improve the performance of the models and minimize the risk of overfitting, the hyperparameters for each model were optimized through a grid search approach with Scikit-Learn’s GridSearchCV (version 0.21.3 for python 3.6) [36]. The combination of hyperparameters that resulted in the lowest mean absolute error (MAE) was chosen for each model based on the results of 5-fold cross-validation.

Machine learning offers a data-driven approach to derive patterns and predictions from large datasets. In this study, we utilized the Gradient Boosting Regressor (GBR), a powerful and widely recognized technique that builds predictive models from an ensemble of simpler models, improving its predictions iteratively [38]. Its efficacy in medical predictions, particularly in radiological data, has been previously documented [43].

Sequential Backward Floating Selection (SBFS) is an optimization technique tailored for feature selection, ensuring the model is not misled by redundant or irrelevant information [44]. By systematically evaluating and eliminating the least contributive features, SBFS refines our dataset to the most predictive features.

**Fig 3:**
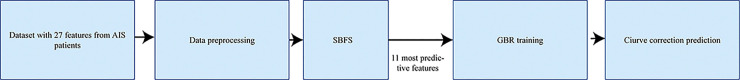
Flowchart illustrating the development of the machine learning model for predicting AIS curve correction.

The success of AVBT surgery is determined by the correction of the major coronal Cobb angle to less than 35°, without the requirement for additional surgical intervention or fusion at the latest follow-up [7–9, 45]. Hence, instead of predicting a single value for curve correction, the model provides a 90% prediction interval. If the interval falls between 0 and 35 degrees, the surgery is considered to have achieved a successful outcome. This diagram in Fig 3 outlines the step-by-step process of data ingestion, preprocessing, feature selection via SBFS, model training using GBR, and eventual curve prediction.

### Deployment of the curve correction prediction model

To ensure the clinical translation of the reported findings, and for the model to be more widely used, a web user-interface app to predict AVBT-based curve correction in AIS patients was created. Model deployment was done using Flask and Heroku, where Flask is a micro web framework to create web applications, and Heroku is a cloud platform to host such web applications.

## Results

The current study analyzed data from 84 (out of 91) pediatric patients that met the inclusion criteria. Seven patients who were excluded had missing clinical or radiographic data. Of these 84 patients, 74 were female and 10 were male, with an average age of 12.2 ± 1.02 years and 12.5 ± 1.96 years, respectively. 82 of the 84 patients had a midterm follow-up at five years or their latest available visit, while the other two patients had follow-ups at 2.5 and one year postoperatively. The follow-up duration ranged between one and 9.25 years, with a median of 5.5 (1.3) years. The collected continuous and categorical features from the patients’ initial visit included in the analysis are presented in Tables 3 and 4, respectively. Table 5 illustrates the curve correction data at the postoperative follow-up visits.

**Table 3:** Description of continuous features at the initial visit for the subjects included in the data analysis.

Features	Mean	SD	Min.	Max.
Major Cobb (°)	48.75	10.96	23.02	76.18
Major Cobb tether(°)	46.47	11.67	11.66	76.18
Lumbar Cobb angle (°)	33.10	11.23	15.96	67.62
Age (in years) at Surgery	12.22	1.13	9	15
Coronal_Balance_preop (mm)	1.15	1.05	0.00	6.54
Shoulder_Balance_preop (mm)	0.96	0.79	0.00	3.42
L4_tilt_preop (°)	9.53	6.75	0.00	26.92
trunk_shift_preop (mm)	1.51	1.21	0.00	5.79
Thoracic_Kyphosis_preop (°)	23.64	11.92	0.22	55.30
Proximal_Thoracic_Kyphosis_preop (°)	10.42	6.53	0.44	30.53
Mid_lower_thoracic_kyphosis_preop (°)	16.88	9.44	0.37	41.30
Thoracolumbar_kyphosis_preop (°)	9.61	7.29	0.08	31.29
Lumbar_Lordosis_preop (°)	70.20	14.41	1.15	102.48
Preop Bending Proximal Thoracic Curve (°)	16.50	9.61	0.55	48.50
Preop Bending Thoracic (°)	19.95	11.08	0.51	46.70
Preop Bending Lumbar (°)	9.53	7.36	0.03	34.00
Flexibility (°)	0.60	0.20	0.03	0.99

**Table 4:** Description of categorical features at the initial visit for the subjects included in the data analysis.

Gender	Male	Female	
	10	74	
Sanders	1	2	3
	1	25	42
	4	5	6
	14	1	1
Major Apex	T7	T8	T9
	2	34	26
	T10	T11	T12
	13	3	1
	L1	L2	L3
	2	2	1
	Min	Max	Mode
UIV	T5	T10	T5
LIV	T10	L3	T12
NIV	4	9	8

**Table 5:** Description of spinal curve magnitude and correction at preop and follow-up visits.

	Preop (initial visit)	First Erect postop	1 yr postop	2 yr postop	5 years postop	Latest postop
Number of subjects	84	84	73	71	40	65
Visit months from surgery	N/A	0.88±0.54	12.04±1.92	24.51±2.65	62.24±8.65	66.39±15.87
		(0–1)	(8–21)	(20–33)	(44–87)	(18–111)
(Mean±SD)						
(Min-Max)						
Age (in years)	12.3±1.14	12.3±1.14	13.3±1.2	14.28±1.2	17.4±1.35	17.72±1.44
(Mean±SD)		(9.2–15.1)	(10.2–16.3)	(11.2–16.9)	(14.2–19.8)	(14.7–23)
(Min-Max)						
Cobb angle (°)	46.47±11.67	24.74±10.98	18.13±10.72	19.39±11.75	20.99±13.08	26.87±12.14
(Mean±SD)		(1.1–55.7)	(0.24–49.6)	(1–52.3)	(0.4–57.3)	(0.8–52.3)
(Min-Max)						
Mean Difference (°)	N/A	21.72±9.83	27.60±12.13	27.01±11.13	21.26±14.10	20.38±14.59
(preop vs. timepoint)						
%Correction	N/A	46±25	59±26	58±25	50±31	40±34

### Most predictive features selected from the SBFS method

The input features used by the Sequential Backward Floating Selection (SBFS) method and their indices are listed in Table 2. The results of the feature selection method are shown in Table 6, where the average mean absolute error (MAE) from a 5-fold cross-validation is presented in descending order for the Gradient Boosting Regressor (GBR) model. Out of the 27 features, the top 11 most predictive features were selected by SBFS are listed in Table 7. These 11 features include: first erect major Cobb angle, age at visit, preop thoracic bending curve, preop proximal thoracic bending curve, shoulder balance, age at surgery, flexibility, preop proximal thoracic curve, Sanders stage, number of instrumented vertebrae, major curve apex.

**Table 6:** Output from SBFS ranked in descending order. MAE of final major Cobb angle correction prediction, CI represents the 95% confidence interval around the computed cross-validation scores.

Features Combination	Average MAE scores	Standard Deviation	(CI) bound
(2, 3, 6, 7, 8, 11, 15, 19, 20, 24, 26)	7.97	1.66	2.13
(2, 3, 6, 7, 8, 11, 19, 20, 24, 26)	7.98	1.65	2.12
(2, 3, 6, 7, 8, 11, 19, 24, 25)	8.00	1.66	2.14
(2, 3, 6, 7, 8, 11, 19, 20, 22, 23, 24, 26)	8.03	1.70	2.19
(2, 3, 6, 7, 8, 11, 19, 24)	8.06	1.61	2.07
(2, 3, 6, 7, 8, 11, 12, 19, 20, 22, 23, 24, 26)	8.09	1.74	2.24
(2, 3, 4, 6, 7, 8, 12, 13, 19, 20, 22, 23, 24, 26)	8.11	1.63	2.10
(2, 3, 4, 6, 7, 8, 12, 13, 14, 19, 20, 22, 23, 24, 26)	8.14	1.56	2.01
(2, 6, 7, 8, 11, 19, 24)	8.15	1.58	2.04
(2, 3, 4, 6, 7, 8, 9, 12, 13, 14, 19, 20, 22, 23, 24, 26)	8.21	1.59	2.04
(2, 3, 4, 6, 7, 8, 9, 10, 13, 14, 19, 20, 22, 23, 24, 25, 26)	8.26	1.61	2.07
(2, 6, 7, 8, 19, 24)	8.26	1.44	1.86
(2, 3, 4, 6, 7, 8, 9, 10, 13, 14, 18, 19, 20, 22, 23, 24, 25, 26)	8.35	1.63	2.09
(2, 3, 4, 6, 7, 8, 9, 10, 13, 14, 17, 18, 19, 20, 22, 23, 24, 25, 26)	8.46	1.66	2.13
(0, 2, 3, 4, 6, 7, 8, 9, 10, 13, 14, 17, 18, 19, 20, 22, 23, 24, 25, 26)	8.48	1.65	2.12
(2, 3, 7, 19, 24)	9.01	1.52	1.95
(0, 2, 3, 4, 5, 6, 7, 8, 9, 10, 13, 14, 17, 18, 19, 20, 22, 23, 24, 25, 26)	7.97	1.62	2.08
(0, 1, 2, 3, 4, 5, 6, 7, 8, 9, 10, 13, 14, 17, 18, 19, 20, 22, 23, 24, 25, 26)	7.98	1.58	2.03
(2, 7, 19, 24)	8.00	1.42	1.83
(0, 1, 2, 3, 4, 5, 6, 7, 8, 9, 10, 12, 13, 14, 17, 18, 19, 20, 22, 23, 24, 25, 26)	8.03	1.56	2.00
(0, 1, 2, 3, 4, 5, 6, 7, 8, 9, 10, 12, 13, 14, 16, 17, 18, 19, 20, 22, 23, 24, 25, 26)	8.06	1.49	1.92
(0, 1, 2, 3, 4, 5, 6, 7, 8, 9, 10, 11, 12, 13, 14, 16, 17, 18, 19, 20, 22, 23, 24, 25, 26)	8.09	1.62	2.08
(2, 7, 19)	8.11	1.48	1.91
(0, 1, 2, 3, 4, 5, 6, 7, 8, 9, 10, 11, 12, 13, 14, 15, 16, 17, 18, 19, 20, 22, 23, 24, 25, 26)	8.14	1.69	2.18
(2, 7)	8.15	1.47	1.89
(0, 1, 2, 3, 4, 5, 6, 7, 8, 9, 10, 11, 12, 13, 14, 15, 16, 17, 18, 19, 20, 21, 22, 23, 24, 25, 26)	8.21	1.89	2.43
(2,)	8.26	1.39	1.79

**Table 7:** Rank and weights of most important features to predict AIS curve correction after AVBT surgery.

Rank	Feature	Importance (weight)
1	First erect major Cobb angle	0.332
2	Age at visit	0.179
3	Preop thoracic bending curve	0.116
4	Preop proximal thoracic bending curve	0.084
5	Shoulder balance	0.075
6	Age at Surgery	0.066
7	Flexibility	0.048
8	Preop proximal thoracic curve	0.042
9	Sanders stage	0.030
10	Number of instrumented vertebrae	0.022
11	Major curve apex	0.007

### Gradient Boosting Regressor (GBR) to predict corrected final major Cobb angle

**Fig 4:**
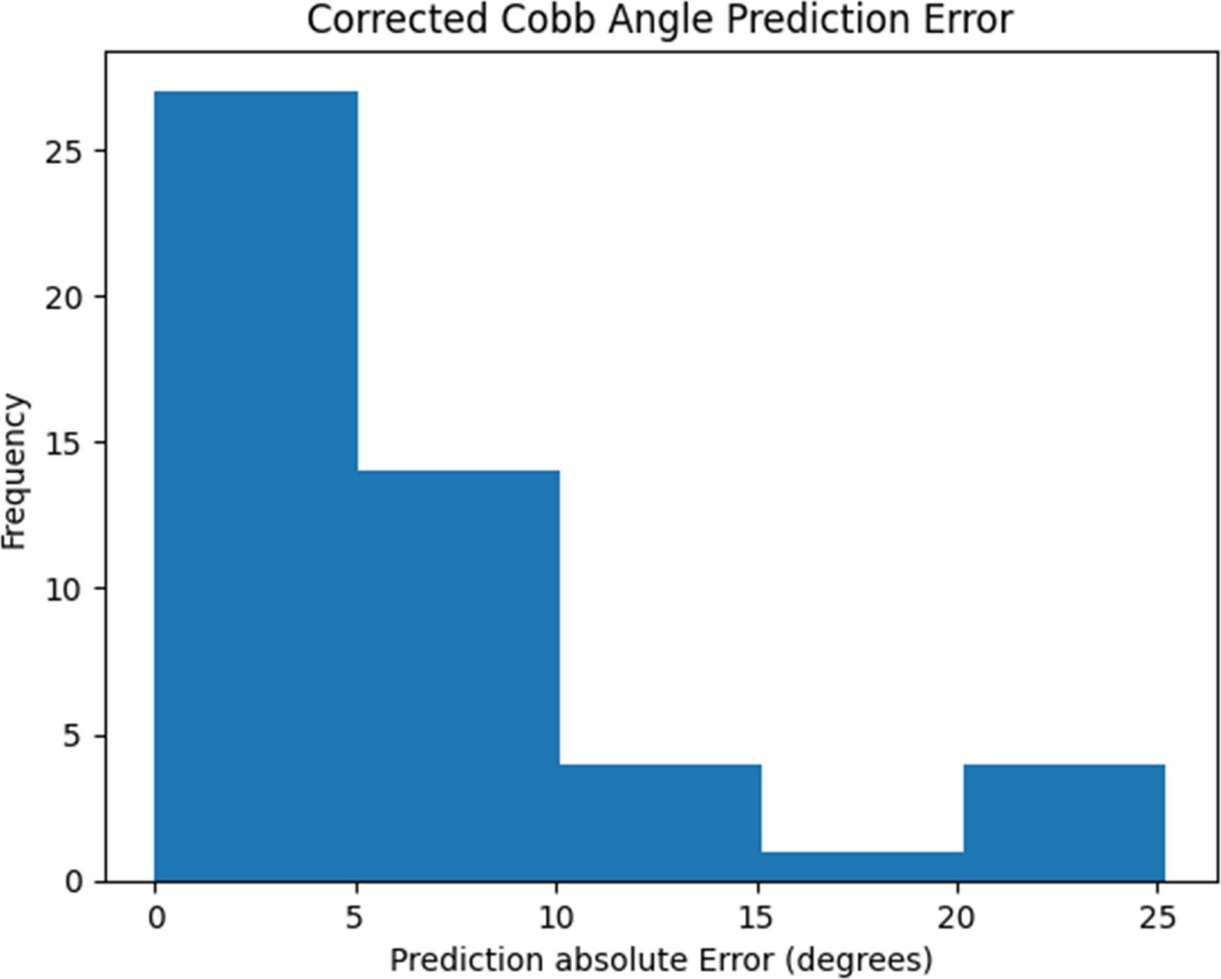
The mean absolute error of the testing dataset predictions using GBR model.

**Fig 5:**
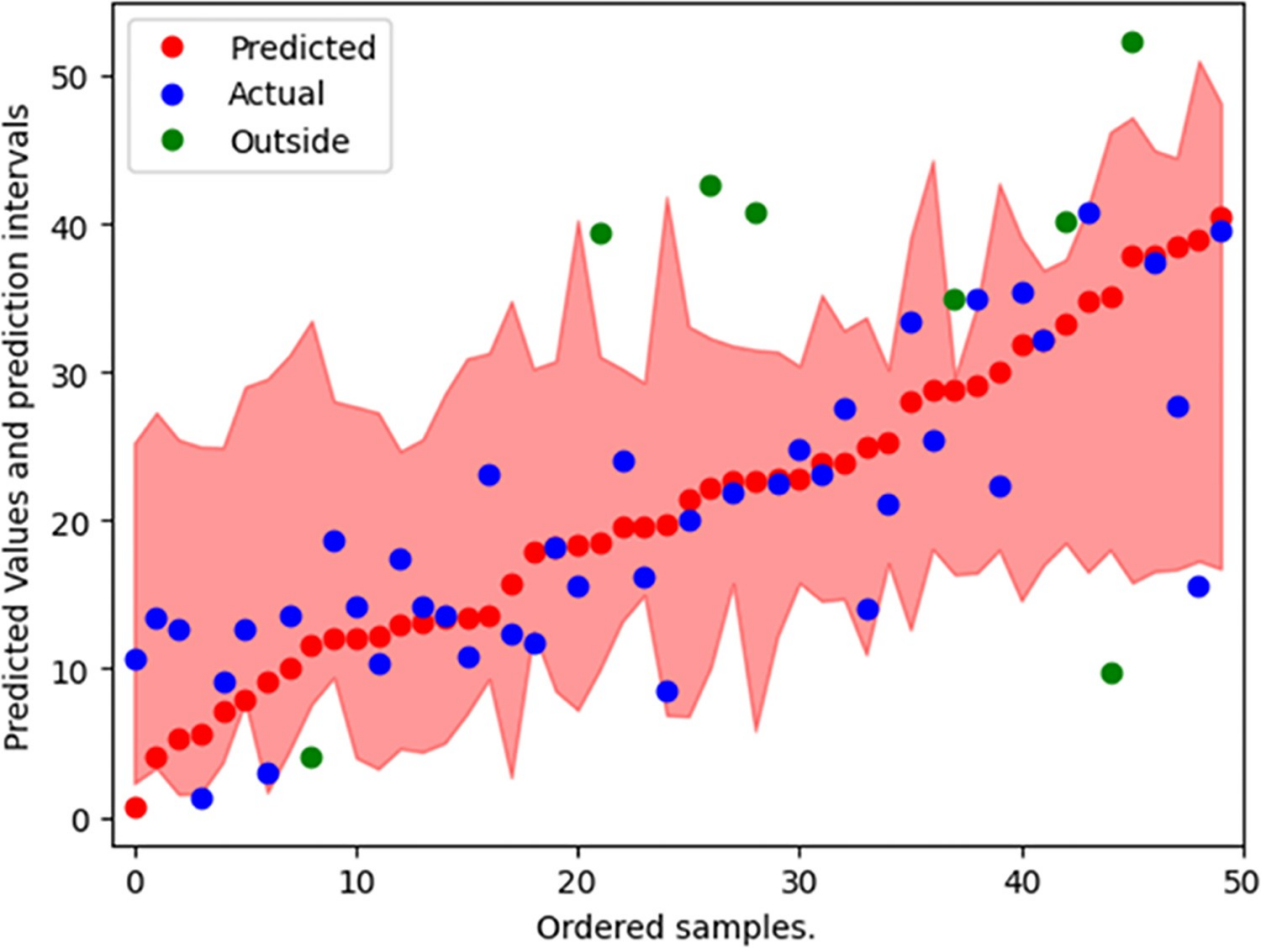
The prediction intervals generated by the GBR model for each observation in the testing dataset.

The range of hyperparameters used to optimize the machine learning models and the selected values are presented in Table 8. The top 11 most predictive features, as selected by the SBFS algorithm, were used as inputs for each of the models. The performance of the models is summarized in Table 9. The GBR model predicted the corrected final major Cobb angle with the lowest MAE, and therefore selected as the method to predict curve correction after AVBT surgery. The testing errors and their frequencies are shown in Figs 4 and 5 shows that 84% of the actual Cobb angle values were found to be within the 90% prediction interval generated by the GBR model.

**Table 8:** Hyperparameters grid values and the selected values for optimized models.

Hyperparameter	Range of values	Selected Value
Random Forest		
n_estimators	[100500]	200
max_features	[17]	‘sqrt’
max_depth	[150]	80
min_samples_split	[211]	3
min_samples_leaf	[111]	1
Gradient Boosting Regressor		
n_estimators	[100500]	200
max_features	[17]	‘sqrt’
max_depth	[150]	70
min_samples_split	[211]	6
min_samples_leaf	[111]	7
Support Vector Machine		
Epsilon	[0,1,10,100,1000]	0
Gamma	[1,0.1,0.001,0.0001]	0.001
Artificial Neural Network		
batch_size	[10,20,30,40,50]	20
epochs	[10,20,30,40,50]	10
Optimizer	[‘adam’, ‘rmsprop’]	adam

**Table 9:** Performance of the ML models in terms of mean absolute error (MAE) of final Cobb angle predictions (degrees) (descending).

Model	Training MAE (°)	Testing MAE (°)
Gradient Boosting Regressor	7.2	6.3
Random Forest	7.3	6.6
Artificial Neural Network	10.2	8.7
Support Vector Machine	10.2	9.3

### Web user interface app for AIS curve correction prediction after AVBT surgery

The web user interface app is publicly available at: https://biomed.drexel.edu/labs/obl/toolkits/predict-curve-correction/.

## Discussion

This is the first study to apply machine learning methods to longitudinal data from a cohort of AIS patients who underwent AVBT surgery to identify a rank ordered list of the most predictive features associated with curve correction post-AVBT. A GBR model was developed and validated using the list of the most important features to predict short- and mid-term curve correction following AVBT surgery. The GBR model predicted curve correction within an acceptable mean absolute error of 6.3 degrees.

The curve correction following AVBT happens in two phases, namely: immediate intraoperative and postoperatively with growth. The immediate intraoperative correction can be estimated using the flexibility rate extracted from supine or fulcrum bending radiographs [34, 46–48]. However, this method is unable to predict the postoperative correction with the growth remaining, which the AVBT leverages through the application of the Heuter-Volkmann principle of growth modulation. The predictability and reliability of spinal growth modulation are largely dependent on a complex interplay of factors. These include patient factors such as preop major Cobb angle, curve flexibility, growth remaining, and the response of the uninstrumented compensatory (i.e., nonstructural) curves. Surgical factors also play a role, including surgical technique, levels selected for instrumentation, number of levels instrumented, and the initial correction obtained [34]. Hence, for this study we collected clinical, surgical, and radiographic features to predict the magnitude of AVBT surgical correction achieved in AIS patients. Using SBFS, eleven prognostic features were selected out of 27 collected features.

The selected predictive feature with the highest rank is the initial correction obtained through AVBT. The initial correction plays a crucial role in the final correction of AIS curves and is responsible for the majority of the total correction achieved [34, 49]. Small to moderate gains in correction can be achieved through ongoing spinal growth, unless the patient has extreme remaining growth [49]. However, the lack of intraoperative radiographs for the patient cohort included in this study necessitated the use of the first erect radiograph as a surrogate for determining the initial correction. It is important to note that intraoperative correction during AVBT may deteriorate by an average of 10° on the first erect spine radiographs [50]. This reduction is the result of the application of gravitational force to the dynamic tether system as the patient stands. Determining the desired initial correction will inform the size of the residual curve following AVBT surgery and optimize AVBT outcomes. In addition to the first erect major Cobb angle, the number of instrumented vertebrae (NIV) was also selected as a predictive surgical parameter in determining curve correction, thereby providing valuable information to assist surgeons in level selection during surgical planning.

The patient’s age at surgery, the patient’s age at the time of the postoperative visit, and the Sanders stage [51, 52] were also selected as most predictive features for curve correction. As AVBT indication criteria include skeletal immaturity [8, 53], substantial growth should remain to obtain correction and avoid undercorrection or overcorrection complications based on the residual curve. Age at surgery and age at visit are correlated with the amount of growth remaining. These features will help with clinical decision making on the timing of surgery. Sanders maturity was ranked 10^th^ (out of 11) most predictive feature for curve correction. As patients with Sanders stage of three (out of six Sanders skeletal maturity stages) are considered as those with the ideal remaining growth for AVBT [14, 52], the majority of the patients in this study cohort had Sanders stage three (50%) followed by Sanders stage two (30%). This, in turn, may have reduced the rank of this feature.

Preoperative curve magnitude (Proximal thoracic and thoracic bending) and curve flexibility are factors used to account for curve stiffness. Silk et al. [34] correlated Fulcrum Flexibility Rate (FFR) with Correction Rate (CR) and assessed the use of FFR to reliably predict residual deformity following Anterior Vertebral Body Tethering (AVBT) surgery. The results of their study demonstrated patients with large and/or stiff curves may exhibit a less favorable initial response to the tethering technique. Stiff curves were found to be preoperatively larger (54° vs 48°) and to have a lower fulcrum flexibility rate (48% vs 79%) compared to flexible curves. Furthermore, significant difference in the correction rate (50% vs 74%) was observed between the rigid and the flexible groups, a factor that could be crucial when assessing a patient’s suitability for AVBT [34].

In Table 5, our cohort data highlights the complex nature of growth modulation post-AVBT. From the first erect measurement to the 5-year follow-up, we observe a decrease of around 3.7 degrees in the mean Cobb angle, suggesting that AVBT has a significant influence on growth modulation, at least in the midterm. However, by the final follow-up, the curve magnitude surpasses the initial first erect value by an average of 2.2 degrees, indicating variability in the long-term effectiveness of AVBT. Several factors could explain this trend. First, the decrease in the Cobb angle up to the 5-year point validates AVBT’s capability in guiding vertebral growth in the desired direction. Second, the subsequent increase in curve magnitude showcases potential differences in individual patient responses to AVBT over extended periods. Lastly, the increase in the Cobb angle by the final follow-up may also be influenced by natural changes in the biomechanics of the growing spine, not just the effects of AVBT.

This study has a few limitations. Although tether tensioning is an important parameter for curve correction, the magnitudes of the applied tether tension were not available for the cohort of AIS patients used in this study. Therefore, tether tension was not included as a feature in the current study. However, the current GBR model predicts intraoperative correction required for successful surgery outcome, which is reported to be less than 35 degrees of Cobb angle at skeletal maturity with no conversion to Posterior Spine Fusion [14, 51–53], combined with curve flexibility, may help the surgeon determine the appropriate amount of the intraoperative tether tension needed. Additionally, the GBR model cannot extrapolate beyond the data values it was trained on [54, 55]. However, expanding the dataset can help address this limitation in future iterations. Moreover, the outcomes of this current study may be affected by institutional bias, and future work could include datasets from other institutions.

## Conclusion

The most predictive features of curve correction using AVBT were identified and rank-ordered. A Gradient Boosting Regression (GBR) model was trained and validated using these selected features, demonstrating a capability of predicting the final curve magnitude within a clinically acceptable range. The current model has potential to serve as a valuable clinical tool, providing insight into the optimal timing of intervention and surgical planning parameters, which may result in improved surgical outcomes and facilitate informed decision-making in patient selection and timing for AVBT surgery.
